# Changes of Serum Ficolin-3 and C5b-9 in Patients with Heart Failure

**DOI:** 10.12669/pjms.37.7.4151

**Published:** 2021

**Authors:** Hongli Li, Fangfang Zhang, Dan Zhang, Xiang Tian

**Affiliations:** 1 Hongli Li, Department of Cardiology, Baoding First Central Hospital, Baoding, Hebei 071000, China; 2 Fangfang Zhang, Department of Cardiology, Baoding First Central Hospital, Baoding, Hebei 071000, China; 3 Dan Zhang, Department of Cardiology, Baoding First Central Hospital, Baoding, Hebei 071000, China; 4 Xiang Tian, Department of Cardiology, Baoding First Central Hospital, Baoding, Hebei 071000, China

**Keywords:** Ficolin-3, C5b-9, Heart failure

## Abstract

**Objectives:**

To investigate the correlation of serum ficolin-3 and C5b-9 with cardiac function and NT-proBNP in patients with heart failure.

**Methods:**

Sixty patients with heart failure admitted to the Baoding First Central Hospital from May 2019 to May 2020 were selected and divided into three groups according to the classification of New York Heart Association (NYHA). Patients with NYHA grade II, III, and IV were included into group A, B, and C, respectively. Among the population undergoing physical examination at the same time, 20 cases with no significant difference in age and gender from the experimental group were selected as the control group (Group-N), and their clinical data were recorded. The serum levels of ficolin-3, C5b-9 and NT-proBNP in each group were detected and compared.

**Results:**

The serum concentrations of ficolin-3 and C5b-9 in Group N were significantly different from those in Group A, B and C (p<0.05), the difference between Group C and Group A and B was statistically significant (p<0.05), there was no significant difference between group A and B (p>0.05). The correlation analysis between serum ficolin-3 and NT-proBNP showed that serum ficolin-3 was negatively correlated with NT-proBNP (r=-0.606, p<0.0001), while the correlation analysis between serum C5b-9 and NT-proBNP showed that serum C5b-9 was positively correlated with NT-proBNP (r=0.499, p<0.0001). According to the etiology of heart failure, patients with heart failure were divided into coronary heart disease (25 cases), dilated cardiomyopathy (15 cases) and others (20 cases). The differences of ficolin-3 and C5b-9 among patients were compared, and there was no statistical difference (p<0.05).

**Conclusion:**

Ficolin-3 was inversely associated with the severity of heart failure, while C5b-9 was positively associated with the severity of cardiac impairment. Both of them have nothing to do with the etiology of heart failure.

## INTRODUCTION

Heart failure (HF) is the common end of a variety of heart diseases,^1^ which seriously threatens human health. It has been shown in a large number of studies that complement exerts an important role in the occurrence and development of HF.^2^-^5^ Ficolin-3 is a promoter of the complement lectin pathway, which can activate the lectin pathway of complement so that the complement system is fully activated, and the terminal complement complex C5b-9 is eventually formed. Studies have shown that the reduction of serum ficolin-3 levels has a close bearing on a variety of cardiovascular diseases.^3^ According to foreign studies on Bulgarian and Norwegian races, ficolin-3 is associated with heart failure,^4^ but few studies have been carried out in China on ficolin-3 in heart failure, and most of the studies on ficolin-3 have focused on autoimmune system and inflammation and other diseases. Instead, most of the studies on ficolin-3 have focused on diseases such as autoimmune system and inflammation.^6^-^8^ In this study, the role of ficolin-3 and C5b-9 in heart failure was studied to explore the relationship between ficolin-3 and C5b-9 and cardiac function in patients with heart failure, so as to provide new methods for the diagnosis and prognosis of heart failure, and to conceive new treatment ideas and schemes for delaying the progression of heart failure.

## METHODS

Patients with heart failure with ejection fraction less than 0.45 admitted to the Department of Cardiology, Baoding First Central Hospital between May 2019 and May 2020 underwent routine echocardiography, and their ejection fraction, E/A value, left ventricular end-systolic diameter, and left ventricular end-diastolic diameter were recorded. The study subjects were determined by medical history, symptoms, signs, and cardiac color Doppler ultrasound results, and were divided into three groups according to the NYHA functional classification: Group-A, Group-B and Group-C. Among them, 20 patients with NYHA-II were divided into Group-A, 20 patients with NYHA-II were included into Group-B, and 20 patients with NYHA-IV were included into Group-C. Among the population undergoing physical examination, 20 cases with no significant difference in age and gender from the experimental group were selected as the control group (Group-N).

### Inclusion criteria

Based on the 2012 European Society of Cardiology Guidelines for the Diagnosis and Treatment of Acute and Chronic Heart Failure:

- Typical symptoms of heart failure;
- Typical signs of heart failure;
- Decreased left ventricular ejection fraction. Patients need to meet the above three criteria to be included.

### Exclusion criteria:

- Patients with acute or chronic inflammatory diseases and immune system diseases;
- Patients with pregnancy, severely impaired liver and kidney function;
- Patients with acute cerebrovascular disease and malignant tumor;
- Patients with acute coronary syndrome, congenital heart disease, and other serious complications. This study was approved by the Ethics Committee of Baoding First Central Hospital, (January 13, 2021) and all the subjects signed the informed consent form.

### Clinical data collection

The clinical data of each group were recorded, including age, gender, blood lipid, NYHA heart function classification, primary disease, smoking history, alcohol history and general clinical data.

### Serological index detection

In the early morning of the next day after admission, 4ml of fasting elbow venous blood was collected from the patients who met the diagnostic criteria and centrifuged for 20min (3000r/min) after standing. The serum was separated and transferred into the freezing tube. After labeling, the samples were frozen at -70 °C. All samples were collected for detection at one time. The human ficolin-3 kit provided by Nanjing Jiancheng Research Institute was used to detect the serum ficolin-3 level by enzyme-linked immunosorbent assay. The sensitivity of the kit: 1.0 ug/ml, strong specificity, detection value range: 0-160 ug/ml. The testing process was operated in strict accordance with the instructions. Serum C5b-9 was measured by ELISA kit of human terminal complement complex C5b-9 provided by Nanjing Jiancheng Research Institute. The sensitivity and specificity: 1.0 ng/ml, strong specificity, detection value range: 0-2000 ng/ml. The testing process was operated in strict accordance with the instructions. NT proBNP was measured by the Department of Laboratory Medicine, Baoding First Central Hospital.

### Statistical Processing

SAS statistical software was used for data processing, and the experimental data was expressed as mean ± standard deviation. T test was adopted for inter-group data comparison, linear correlation analysis was used to analyze the correlation between the two groups, and analysis of variance was used for the comparison between the two groups. P<0.05 indicates a statistically significant difference.

## RESULTS

No significant difference could be seen between the control group (normal healthy group) and the experimental group (heart failure group) in terms of average age, gender, hypertension, diabetes, smoking, drinking and other general clinical data, and there was no statistical significance. (p>0.05) (Table-I).

**Table I:** Basic information of subjects.

	*Group-N*	*Group-A*	*Group-B*	*Group-C*
The number of cases	20	20	20	20
Age	61±12.53	64±11.46	62.65±16.22	64.35±12.22
Gender (male/female)	10/10	9/11	10/10	10/10
Smoking (%)	6(30%)	5 (25%)	5 (25%)	6 (30%)
Hypertension (%)	— —	10 (50%)	11 (55%)	12 (60%)
Diabetes (%)	— —	7 (35%)	5 (25%)	8 (40%)
CHOL (mmol/l)	4.19±0.8	3.96±1.20	3.72±1.11	4.35±1.21*
TG (mmol/l)	1.63±0.53	1.34±0.75	1.18±0.48*	1.06±0.31*
HDL (mmol/l)	0.91±0.16	1.0±0.29	0.93±0.21	1.01±0.27
LDL (mmol/l)	2.59±0.68	2.61±0.88	2.57±0.93	3.19±1.01*
ALT (u/l)	21.84±8.01	24.7±8.08	30.21±28.34	34.78±32.80
AST (u/l)	22.66±14.21	20.15±6.38	38.67±61.31	40.71±40.8
Cr (umol/l)	59.37±7.65	56.5±10.06	71.07±35.48	80.62±66.06
LVDD (mm)	— —	54.35±6.41*	59.9±6.33*	67.5±12.16*
Peak E (m/s)	— —	0.94±0.42	0.89±0.47	0.88±0.24
Peak A (m/s)	— —	0.69±0.21	0.63±0.26	0.65±0.31
LVEF(%)	— —	41.3±4.55	37.1±6.99	34.3±8.65*

***Note:***

* p<0.05.

**Fig.1:**
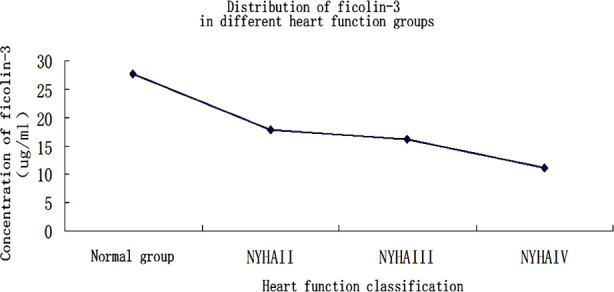
Concentration of serum ficolin-3 in each group.

The concentration of serum ficolin-3 in Group-N was 27.760±14.835 ug/ml, that in Group-A was 17.896 ± 1.308ug /ml, that in Group-B was 16.096±0.94ug/ml, and that in Group-C was 11.197± 1.899 ug/ml. In terms of pairwise comparison between groups, Group-N was statistically different from groups A, B, and C (p<0.05), Group-C was statistically different from Group-A and Group-B (p<0.05), but there was no statistical difference between Group-A and Group-B (p>0.05). (Table-II and Fig.1)

**Table II:** Concentrations of experimental indexes in each group.

	*NT-proBNP(pg/ml)*	*Ficolin-3(ug/ml)*	*C5b-9(ng/ml)*
Group-N	— —	27.760±14.835	87.397±54.011
Group-A	579.800±232.091	17.896±1.308	233.759±17.659
Group-B	3944.950±1126.130	16.096±0.94	320.793±34.956
Group-C	16572.200±10775.192*	11.197±1.899*	658.657±366.514*

***Note:***

* p<0.05.

**Fig.2:**
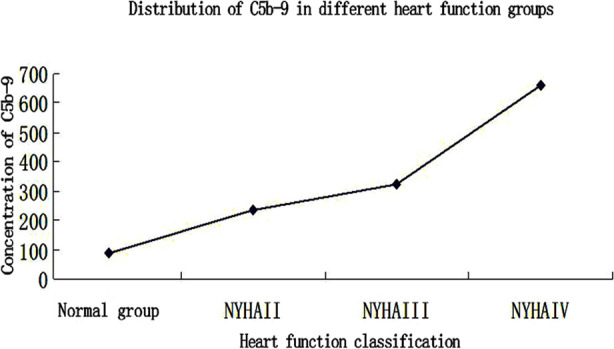
Concentration of C5b-9 in each group.

The concentration of serum C5b-9 in Group-N was 87.397±54.011 ng/ml, that in Group-A was 233.759±17.659 ng/ml, that in Group-B was 320.793±34.956 ng/ml, and that in Group-C was 658.657±366.514 ng/ml. In terms of pairwise comparison between groups, Group-N was statistically different from groups A, B, and C (p<0.05), Group-C was statistically different from Group-A and Group-B (p<0.05), but there was no statistical difference between Group-A and Group-B (p>0.05). (Table-II and Fig.2)

**Fig.3:**
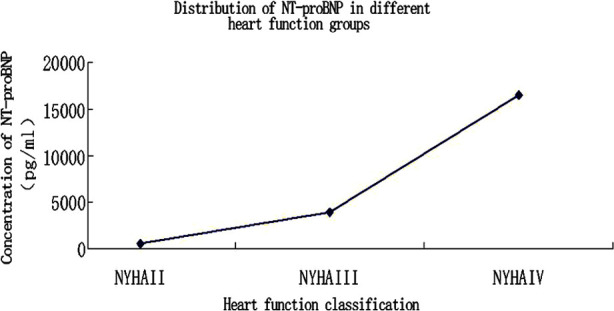
Concentration of serum NT-proBNP in the experimental group.

**Fig.4:**
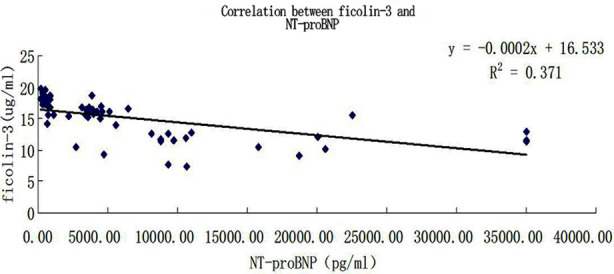
Correlation analysis between ficolin-3 and NT-proBNP.

**Fig.5:**
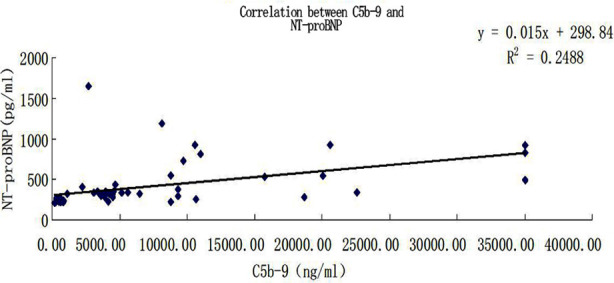
Correlation analysis between C5b-9 and NT-proBNP.

The concentration of serum NT-proBNP in Group-A was 579.800±232.091 pg/ml, that in Group-B was 3944.950±1126.130 pg/ml, and that in Group-C was 16572.200±10775.192 pg/ml. In terms of pairwise comparison between groups, Group-C was statistically different from Group-A and Group-B (p<0.05), but there was no statistical difference between Group-A and Group-B (p>0.05), as shown in Table-II and Fig.3.Ficolin-3 was significantly negatively correlated with NT-proBNP (r=-0.606, p<0.0001), as shown in Fig.4.C5b-9 was significantly positively correlated with NT-proBNP (r=0.499, p<0.0001), as shown in Fig.5.

According to the etiology of heart failure, heart failure was divided into coronary heart disease group, dilated cardiomyopathy group and other, and the relationship between ficolin-3, C5b-9 and NT-proBNP was compared among the three groups, as shown in Table-III.It can be seen from Table-III that there was no statistical difference between the groups, that is, ficolin-3 and C5b-9 have nothing to do with the etiology of heart failure, but are only linked to the severity of heart failure.

**Table III:** Comparison between different etiologies.

*Etiology*	*Ficolin-3(ug/ml)*	*C5b-9(ng/ml)*	*NT-proBNP(pg/ml)*
Coronary heart disease (n=25)	14.92±3.55	354.07±168.28	4510.29±8106.68
Dilated heart disease (n=15)	15.07±2.81	481.63±402.56	3875±6446.19
Other (n=20)	15.22±3.08	412.85±288.26	2053.21±1745.90
F value	0.05	0.95	0.62
P	0.9514	0.3927	0.5413

## DISCUSSION

Cardiomyocytes in patients with heart failure are characterized by a variety of death mechanisms, including necrosis, apoptosis, tumor and autophagy, etc., which are related to complement activation.^9^-^11^ The relationship between complement activation and heart failure is further supported by a study by Prohászka et al.^4^ According to this study, after long-term circulatory support, the deposition of the terminal complement complex C5b-9 in the cardiomyocytes of heart failure increased significantly, and C5b-9 leads to cardiomyocyte necrosis via various mechanisms^12^ and aggravates heart failure.

NT-proBNP is the gold standard for the diagnosis of heart failure, and can be used to determine the prognosis of heart failure.^13^ In this experiment, the correlation analysis between ficolin-3 and NT-proBNP shows that ficolin-3 is negatively correlated with NT-proBNP, indicating that ficolin-3 is likely to be used as a new index for the prognosis of heart failure. Ficolin-3 is easier to determine in serum, and the determination method is constantly simplified. Moreover, ficolin-3 may be widely used in heart failure like NT-proBNP in the future with more and more in-depth and extensive research on ficolin-3.

It is also shown by the results of this study that C5b-9 is significantly positively correlated with NT-proBNP in patients with heart failure (p<0.0001), indicating that complement activation exists in patients with heart failure and is involved in the continuous progress of heart failure. This result is consistent with the theory of the role of complement in heart failure^14^ and demonstrates the positive role that complement activation plays in the progression of heart failure. Based on this, complement may open up a new field for the study of methods to delay heart failure, and become a new target for the treatment of heart failure.

### Limitations and Prospects of the study

Ficolin-3 and C5b-9 have been proved to be correlated with heart function in patients with heart failure in this experiment, but fewer cases were included in this experiment. At the same time, studies odoin the specific action pathways and mechanisms of ficolin-3 and C5b-9 have not yet been carried out, and only macroscopically, the concentration is taken as the measurement standard and the object of comparison. Therefore, further research needs to be carried out in this aspect to clarify the action pathways and mechanisms of ficolin-3 and C5b-9.

### Authors’ Contributions:

**HL & FZ:** Designed this study and prepared this manuscript, and are responsible and accountable for the accuracy or integrity of the work.

**DZ:** Collected and analyzed clinical data.

**XT:** Significantly revised this manuscript.
